# Progress in Fevers

**Published:** 1900-11-10

**Authors:** 


### PROGRESS IN FEVERS.

**Typhoid.**—An outcry has been raised about the epidemic in South Africa, as if it were an unprecedented occurrence; but a similar one took place in the Spanish-American War. Every regiment in the American army was attacked within two months after moving into camps. The results [1] showed once more that it is neither due to miasma nor putrefaction, but to a specific germ transferred from the excreta. Water supplies were rarely affected; but flies, dust, and personal contact carried the infection from man to man; not even gastric troubles could be shown to increase the liability to the disease which attacked a fifth of the army. As the source of infection in each camp was traced, Vaughan holds that there is no ground for thinking that it was due to an evolution of the colon or other bacilli due to fatigue or filth.[2] Vincent [3] lays stress on the need of precautions against it in all wars, especially as regards the water, camping grounds, and food, and the elimination of young and sickly men. The results of Professor Wright's inoculation in India were recorded in a recent number. In Ladysmith 1,700 were inoculated out of 10,500, and the attacks of typhoid in the two groups were 1 in 48, and 1 in 7. Moreover the inoculations were usually performed once only instead of twice.[4] However, later and more exact statistics are anxiously awaited, as it was stated that 10 per cent. of the whole army in Africa—220,000 men—were attacked, and that the mortality up to July was 21 per cent. This mortality is, of course, much higher than that of hospitals in this country, which may be taken as about 17 per cent., while the bathing system shows only 7 or 8 per cent. for enormous numbers. Yarr, at Bloemfontein,[5] found a small reduction of mortality of about 2 per cent. in the once inoculated patients, but other reports are conflicting.

*The Ehrlich Diazo Test.*—Arneill [6] writes strongly of the value of this reaction after examining the urine of nearly 800 patients. It occurs most frequently in typhoid. Indeed, if absent after the first week there is a strong presumption against a diagnosis of typhoid. In 1,223 cases under different observers the reaction was seen in 1,076. Again in tuberculosis it is often seen, and here the augury is very bad. Indeed, cases where it is found for several days in succession may be considered to be far advanced, though the physical signs may be slight. The writer insists on the careful performance of the test by the addition of a little ammonia to a mixture of equal parts of the urine and of the combined solution. At the junction of the ammonia and of the mixture, if the true reaction is present, a definitely red zone is formed, and when shaken a pink foam is produced. Unless these points are observed exactly the test is entirely useless. W. G. Savage [7] in discussing the Widal test, refers to the sedimentation method. This is carried out in glass tubes drawn out to a point. Blood is taken in another pipette, the serum poured off separately, and diluted to 1 in 25. This diluted serum is mixed with equal quantities of typhoid broth in a watch-glass and drawn up into a tube. In 24 hours the upper part is quite clear; white plugs of bacilli clumped together fall towards the bottom. With non-typhoid serum a uniform turbidity remains throughout. The great advantage of the test is that it does away with the use of a microscope and requires no watching.

**Complications.**—Cystitis may last an indefinite time when caused by Eberth's bacillus, and as noticed by Horton Smith may disseminate the disease all the while. T. A. Brown [8] gives some astounding instances. Two patients showed virulent bacilli in the urine three and five years after the fever, and the second case still retained them two years later. In another instance of typhoid cystitis the writer believes that the bacilli must have been introduced by an unclean catheter, for the patient had no symptoms since her attack of typhoid 35 years before until after a cystoscopic examination and hysterectomy. However, no source of infection for the catheters could be traced, and the bacilli were certainly typhoid and answered the most exact tests. The gall bladder may also retain them for long periods. Thus the same writer quotes a case where they were found seven years after the fever. They seem to be usually present during an attack. Chiari found them in nineteen cases out of twenty-two, and Camac has collected 115 instances, while Von Dungern and Droba found them 14 and 17 years after the fever, where biliary troubles had been set up in the interval.[9] Not only may they lead to cholecystitis and abscesses, but it has been suggested that clumps of bacilli may form the nucleus of gall stones. F. W. Russell [10] refers to an interesting paper by J. Tapie on *osteo-myelitis* in typhoid. The symptoms appeared quite suddenly in one patient during convalescence, but generally the course is very slow and gradual. Such swellings may (1) undergo resolution; (2) persist indefinitely; (3) or go on to suppuration. The tibia is affected in 86 per cent. of the cases; the carpus, femur, humerus, ribs, and sternum are also often affected. The bacillus is not always to be found, and the condition may be mistaken for a syphilitic or tubercular one. Unless suppuration occurs Tapie advises non-interference. Brice Poole met with nine instances of periostitis in the Maidstone epidemic, but in none did suppuration take place. Yarr [11] gives an instance of bilateral *gangrene* in a convalescent at Bloemfontein. The heart was normal though weak, the temperature subnormal, and after some pain in one leg both legs became icy cold and without feeling. The right femoral artery was found pulseless and hard up to Poupart's ligament, and the left was blocked about the middle of the thigh. Death occurred in a few days. In a second case[12] the penis was affected, in another the cheek. Keane has collected 47 instances of gangrene in the trunk, head, and neck. *Desquamation* has been noted by Remlinger[13] in many adults as well as in children. It is generally limited to the trunk, and appears when the temperature is falling. He regards it as due to a trophic lesion rather than to sudamina, and remarks that it is sometimes accompanied by alopecia. *Tachycardia* was found by C. Burland[14] in a large proportion of 265 military convalescents, though ordinarily a slow pulse may be expected at that stage. The pulse was under 70 in less than 2 per cent., and 100 or more in 19 per cent., and he suggests that the tachycardia was due to the heavy marching and irregular feeding of a soldier's life. P. C. Fenwick[15] adds that the only cases with slow pulse were in men who had escaped these fatigues, but he would refer the cases of rapid pulse to sympathetic irritation arising in the superior mesenteric plexus. An instance of so-called *typhoid spine* has been again observed by Lovetta Withington.[16] The pain and rigidity lasted for about twelve months, and for part of the time certain vertebral spines were unduly prominent. The affection has been regarded as a neurosis, but it is now suggested that it is due to a specific periostitis, which is not improbable.

**Treatment.**—Andus[17] holds that intestinal antiseptics while unable to destroy the bacillus do prevent distension and undue fermentation. Calomel in the early stages, salol, and turpentine, and large quantities of water by the mouth are among the best remedies for these conditions. In the Philadelphia hospitals, under *cold bathing*, 1,904 cases showed a mortality of only 7·5 per cent., which exactly coincides with the results of Hare at Brisbane. Wilson and Salinger[18] think that under this treatment hæmorrhage is not diminished, though perforation and other complications are. Albuminuria seems to be increased in frequency, but usually passes away during the illness. They give calomel in early stages, and continue the baths even during convalescence, allowing the milder cases to walk to a stationary bath every three hours if the temperature is 101·4. The lowering of temperature is not the main object, but the constant stimulation of physiological processes and the removal of the effects of the disease. They hold that the treatment should be commenced as soon as possible. Kramer[19] thinks that pyrexia is useful, and gives no antipyretics, but calomel followed by naphthaline and salol, and a more free and nourishing diet than is usually allowed, including eggs, starch, and some form of oatmeal. D. E. English[20] recommends carbolated camphor as an intestinal antiseptic. As it will not mix with water three drops are put into a capsule and swallowed immediately. This dose may be repeated every two hours for a day or two, and then at longer intervals. Larger doses of carbolic can be taken in this way than otherwise. The substance is formed by mixing one part of carbolic acid crystals and three of camphor, but an excess of camphor should be present on account of evaporation. In these proportions the carbolic loses its caustic qualities. The writer gives only meat juice for the first two weeks, with water ad libitum; in the third week milk with salt and eggs beaten up. J. Barr[21] insists that milk should always be diluted, and would add to it milk—sugar, and common salt. If curds appear in the stools milk should be peptonised. Water should be given freely, peptonised bread and milk, Benger's Food, meat juices, raw eggs, butter, and cream may be added. He thinks alcohol is generally useless, that it is not a cardiac tonic, though in collapse a few spoonfuls of brandy may be given, and champagne in severe vomiting. He keeps a cold compress over the abdomen over which is a sheet supported on a cradle. The feet and hands are kept warm by wraps and bottles. In severe cases he allows a stream of water at 80° to flow over the patient lying in a hammock, the legs and thighs being kept in dry blankets. If there is much indican in the urine cold water enemata with permanganate may be given daily. Horton Smith's[22] views on the pathology of typhoid, and especially on the danger of infection through the urine, are well worth remembrance He points out that there is no evidence that the bacilli can multiply outside the body, and that as the blood is a bad medium for their growth they rapidly pass from it into some viscus or are destroyed in all but the worst cases. They probably do not exist in the sweat, and the fæces cease to contain them after the first few weeks. They may sometimes be present in the expectoration, while in one patient out of four the urine contains them in very large numbers, which may remain there for an indefinite period. Such urine is generally turbid, and gives a curious "shimmer" when shaken in the light. They are rarely present for the first week or two, and the cystitis when it exists is probably due to a stray bacillus having been carried into the bladder and multiplying there. A rapid cure of this condition may be effected by antiseptic injections, or even more easily by urotropine in 8 or 10 grain doses three times daily, given by the mouth. He would in private patients give the drug as a matter of routine to prevent further infection of other persons, and finds no bad effects from doing so. It appears clear that the urine is a great source of danger, and one rarely recognised, especially as the bacilli can live and multiply in the bladder for years after the patient seems to be perfectly well.

[1] Philadelphia Med. Jour., June 9. [2] New York Med. Jour., June 9. [3] Brit. Med. Jour. Inter. Congress, Sept. 8. [4] Lancet, July 14. [5] Jour. Trop. Med., Aug. [6] Amer. Jour. Med. Sci., March. [7] Clin. Jour., May 2. [8] Med. Rec., Mar. 10. [9] Brit. Med. Jour. Ep., June 16. [10] Birmingham Med. Rev., July. [11] Jour. Trop. Med., Aug. [12] Idem., July. [13] Brit. Med. Jour., June 23. [14] Lancet, July 28. [15] Lancet, Aug. 11. [16] Birmingham Med. Rev., July. [17] Mercks Archives, May. [18] Brit. Med. Jour, Mar. 31. [19] Med. Press, May 2. [20] Med. Rec., June 30. [21] Lancet, Sept. 29. [22] Brit. Med. Jour., Apr. 7.

